# Prevalence and associated factors of risky sexual behaviors among undergraduate students in state universities of Western Province in Sri Lanka: a descriptive cross sectional study

**DOI:** 10.1186/s12978-018-0546-z

**Published:** 2018-06-04

**Authors:** Upuli Amaranganie Pushpakumari Perera, Chrishantha Abeysena

**Affiliations:** 1 Postgraduate Institute of Medicine, University of Colombo, 36/1, Naiwala, Essalla, Veyangoda, Sri Lanka; 2 Department of Public Health, Faculty of Medicine, University of Kelaniya, Kelaniya, Sri Lanka

**Keywords:** Attitudes, Knowledge, Reproductive, Sexual, Undergraduates

## Abstract

**Background:**

Risky sexual behaviors (RSB) are becoming an important problem all over the world. RSB are defined as behaviors leading to sexually transmitted diseases and unintended pregnancies. The objective of this study was to determine the prevalence and associated factors of RSB among undergraduate students in state universities of Western Province in Sri Lanka.

**Methods:**

A descriptive cross sectional study was conducted on1575 second and third year undergraduates using stratified cluster sampling of the selected universities. A pretested self-administered questionnaire was used to assess socio-demographic, knowledge attitudes and behavior on reproductive health. RSB was defined as reporting of one or more following behavior/s; having more than one sexual partner, use of alcohol or inability to use condom or other contraceptive methods in sexual activities. The results were expressed as prevalence and its 95% confidence interval (CI) of RSB. Multiple logistic regression was performed ascertain the association between RSB and possible associated factors. The results were expressed as adjusted odds ratios (AOR).

**Results:**

Prevalence of RSB in last 1 year and 3 months periods were 12.4%, (95% CI: 11.8–13.1) and 12.1% (95% CI: 11.5–12.7) respectively. The significantly associated risk factors for RSB were, attended night clubs in last month (AOR = 3.58, 95% CI: 1.29–9.88), alcohol consumption within last 3 months (AOR = 2.67, 95% CI: 1.87–3.80) and good knowledge on condoms (AOR = 2.82, 95% CI: 1.94–4.10). Those who thought religion was very important to their lives (AOR = 0.68, 95% CI: 0.48–0.95) was a protective factor.

**Conclusions:**

Alcohol consumption and attending night clubs were associated with RSB. Necessary measures should be taken to reduce risk behaviors within university to reduce RSB.

## Plain English summary

Risky sexual behaviors (RSB) are defined as behaviors leading to sexually transmitted diseases and unintended pregnancies. Currently about 100,000 undergraduate students studying at state universities in Sri Lanka who may be at risk of practicing RSB. This study seeks to determine the prevalence of such behaviors among this group of students.

Data were collected from second and third year undergraduate students with a questionnaire on sexual behaviors, other risk behaviors, knowledge and attitudes on sexual and reproductive health. Of students surveyed 12.4% were found to practice RSB within the last 1 year period. Several other behaviors such as alcohol consumption within the last 3 months, attending night clubs in last month and those with good knowledge on condoms were associated with RSB. Those who thought religion was very important to their lives were less likely to practice RSB. Suggestions were made to take necessary steps to minimize alcohol consumption within university and outside, to discourage night clubs attendance by facilitating more recreational activities and to promote religious activities.

## Background

Risky sexual behaviors (RSB) are becoming an important problem all over the world. The Centers for Disease Control and Prevention (CDC) defines RSB as Sexual behaviors leading to unintended pregnancies and sexually transmitted infections (STI) include Human immuno-deficiency Virus (HIV) and acquired immuno-deficiency syndrome (AIDS) [1]. It includes having multiple sexual partners, having sex without using a condom or other contraceptive method. In addition to that, several authors have included the following factors in to their definition of RSB: initiation of first sex at early age before 18 years [2, 3], sexual activity done under the influence of alcohol and anal intercourse [4], sexual violence and transactional sex [3] and paid sex [5].

There is limited literature on sexual practices among various population groups in Sri Lanka. The prevalence of risk behavior among adolescents and young adults was higher than the expected level by parents and teachers [6–8]. Global prevalence studies including other Asian countries would give a better estimate of considerably higher RSB in undergraduates [9, 10]. There is a vast amount of literature on undergraduates’ RSB in African countries indicating that higher prevalence of RSB among them ranging from 7 to 47% [11–17].

Known socio-demographic and economic risk factors associated with RSB are male sex [11, 12, 18], smoking [8, 20], night club attendance [11] and alcohol use [8, 9, 19–21]. In contrast, having a good relationship with friends, peers and parents [9], as well as religiosity [12, 22] have been found to be a protective measure against RSB.

There are more than 105,000 youths studying in universities in Sri Lanka where the majority are not in a relationship [23]. University life is a shift towards greater freedom from family and school backgrounds for most of them. It provides an opportunity to practice new friendships, social mixing and consequently to engage in risky behaviors including RSB [9]. The findings of this study could potentially support to develop programs to reduce RSB and to improve the knowledge and practices via the existing system of tertiary education. Therefore we conducted this study to determine the prevalence and factors associated with RSB among undergraduates in the state universities of the Western Province in Sri Lanka.

## Methods

### Study design

An institution-based descriptive cross-sectional study was conducted in four state universities in the Western Province of Sri Lanka (Univeristy of Colombo, University of Sri Jayewardenepura, University of Kelaniya and University of Moratuwa), representing around 17% of total undergraduates enrolled in state universities in the country.

### Study population

The study population was undergraduate students studying in second and third years which were 18,280 in number [23]. Undergraduates from foreign countries and clergymen undergraduates were excluded. First year students were excluded as they are new to the environment. So their risk behaviors may not be due to as the same factors as second and third year students. Fourth and fifth year students were excluded as these advanced years are not conducted in every course. Exclusion of foreign students was done due to their different socio-cultural background.

### Sample size and sampling technique

Calculated sample size was 1314 with expected prevalence of 13% of heterosexual intercourse without condoms among unmarried, out of school adolescents [7], 1.96 Z value, 3% of precision and a correction for design effect of 2.45 [24, 25] and 10% of non-respondents.

**Fig. 1:**
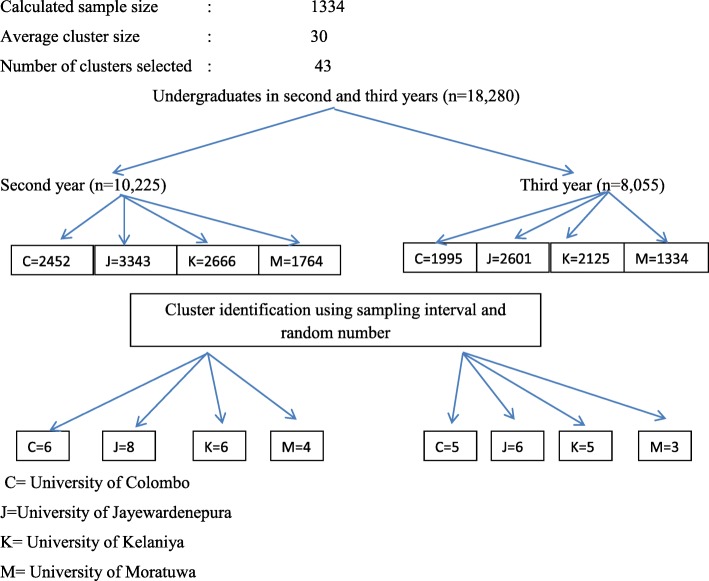
Schematic presentation of sampling technique

A multistage cluster sampling technique with probability proportional to size (PPS) was used to select a representative sample of undergraduates (Fig. 1). A cluster was defined as a tutorial group or a whole batch according to the structure of the selected undergraduates group. The average cluster size was considered as 30. Then we allocated the clusters for each academic year and university according to the proportion of undergraduates. Undergraduates were stratified according to their respective university and academic years and academic streams. Finally clusters were identified within each stratum based on PPS according to the number of students in each university and academic year.

### Data collection

Data were collected on socio-demographic factors, other risk behaviors including consumption of alcohol, smoking and using narcotic drugs, sexual behavior and knowledge and attitudes in selected aspects of reproductive health (RH) among undergraduates. Sri Lanka Behavioral Surveillance Survey questionnaire [26], Youth risk behavior surveillance questionnaire used by CDC [1] and Illustrative Questionnaire for Interview Surveys with Young People [27] were used as guides to develop the questionnaire.

The validity of the questionnaire was ensured by assessing the judgmental validity which included face, content and consensual validity. Validity was determined by assessing the agreement of the experts on whether or not the conceptual definition has been used appropriately in the tool. A multi-disciplinary panel of experts in the fields of public health, reproductive health and language was used for assessment of validity. The questionnaire was forward-translated into Sinhala and back-translated to English. Data were collected using a pre-tested self-administrative questionnaire after obtaining permission. The filled questionnaires in an envelope were collected by trained research assistant. The data collection procedure was supervised by the first author. Informed written consent was obtained from the participants. Ethical clearance was taken from the Ethical Review Committee, Faculty of Medicine, University of Kelaniya.

Knowledge on some aspects of RH was measured with statements on unsafe abortions, contraceptives including condoms, STIs including HIV/AIDS and sexual and RH rights. Attitudes on RH was measured with statements on contraceptives, condoms and HIV/AIDS which contained five responses; strongly agree, agree, neutral, disagree and strongly disagree.

RSB was defined as reporting one or more following behavior/s; having more than one sexual partner, alcohol use with sexual activities, inability to use condom to prevent STI in sexual activities with commercial sex workers or non-commercial partners, unable to use contraceptive methods to prevent unintended pregnancy in sexual activities with commercial sex workers or non-commercial partners. RSB was defined as for the last 1 year and the last 3 months separately.

### Data analysis

Data were analyzed with Statistical Package for Social Science (SPSS) (Version 16). Prevalence and 95% confidence interval (CI) of RSB were calculated for last 1 year and last 3 months periods separately. Sex specific RSB and university specific RSB was calculated for last 3 months period. To ascertain the association between RSB and other variables binary logistic regression was performed. Variables with *p* value less than or equal to 0.20 at binary were entered into multiple logistic regression analysis. Hosmer Lemshow goodness of fit with backward elimination was used to test for model fitness. Variables with p value of < 0.05 at multiple regression were considered as statistically significant. The results were expressed as Odds Ratio (OR) and adjusted odds ratios (AOR).

## Results

### Background information

The study sample consisted of 1575 undergraduates from four state universities in the Western Province. Majority of respondents were females (*n* = 926, 58.8%) and unmarried (98.5%, *n* = 1551). Mean age was 23 (SD = 0.9) years. The sample consisted more Sinhalese (94.1%, *n* = 1480) than non-Sinhalese and Buddhists (89.8%, *n* = 1414) than non-Buddhists. Socio-demographic factors are shown in Table 1.

**Table 1:** Socio-demographic characteristics among the undergraduates

Characteristics		Frequency	Percentage
Sex	Male	645	41.0
	Female	926	58.8
Age (in years)	21	20	1.3
	22	449	28.5
	23	696	44.2
	24	307	19.5
	25	83	5.3
	26	11	0.7
Academic year	Second	930	59.0
	Third	645	41.0
University	Kelaniya	339	21.5
	Sri Jayewardenepura	576	36.6
	Colombo	353	22.4
	Moratuwa	307	19.5
Nationality	Sinhalese	1480	94.1
	Tamil	58	3.7
	Muslims	33	2.1
	Other	1	0.1
Religion	Buddhism	1414	89.8
	Roman Catholic	65	4.1
	Non Roman Catholic Christian	6	0.4
	Islam	45	2.9
	Hindu	39	2.5
	Other	3	0.2
Residence	Own home	453	28.8
	Relative’s house	36	2.3
	University hostel	411	26.1
	Boarding place	671	42.6
Academic streams	Art	516	32.8
	Commerce	424	26.9
	Bio Science	248	15.7
	Mathematics	387	24.6
Type of School	Boys’	262	21.0
	Girls’	345	27.6
	Mixed	642	51.4
Marital status	Single	1551	98.5
	Married	13	0.8
	Divorced	1	0.1
	Separated	1	0.1
	Living together	5	0.3

### The prevalence of RSB

The prevalence of RSB among undergraduates were 12.4% (*n* = 196;, 95% CI: 11.8–13.1) and 12.1% (*n* = 190, 95% CI: 11.5–12.7) for last 1 year and last 3 months respectively. The highest percentage (16.8%, *n* = 57) of undergraduates with RSB was in University of Kelaniya followed by University of Sri Jayewardenepura (12.5%, *n* = 72). Males (19.1, 95% CI 16.1–22.2) had more RSB than females (7.2, 95% CI 5.5–8.9) (*p* < 0.001).

### Sexual behavior of the participants

In the study sample 21.2% (*n* = 334) reported ever having had sexual exposure. Males (32.7%, *n* = 211) had more ever sexual exposure than females (13.1%, *n* = 122) (*p* < 0.001). Majority of undergraduates (63.9%, *n* = 205) who ever had sexual exposure experienced their first intercourse after 20 years of age. More females (75%, *n* = 87) than males (57.8%, *n* = 118) had their first sexual exposure before the age of 20 years (*p* = 0.002). Out of 334 undergraduates had sexual exposure ever, 3.3% (*n* = 11) stated that their first sexual experience was a forced sex. Majority (75.4%, *n* = 252) of the respondent’s first sexual partner was a girl/boyfriend.

Out of 334 with had ever sexual exposure only 18% (*n* = 60) of the respondents had used condoms in first sexual intercourse while only 1.5% (*n* = 5) of the respondents had used alcohol in first sexual intercourse. Out of all undergraduates, 13.7% (*n* = 216) had more than one life time sexual partners.

Percentage of undergraduates who had sexual intercourse within last 1 year was 87.7% (*n* = 293) out of 334 respondents who ever had sex. Majority of them had one sexual partner irrespective of whether they have engaged in sexual intercourse in last 1 year or last 3 months. Ninety four (5.96%) and 79 (5.01%) respondents had multiple sexual partners within last 1 year and 3 months respectively. The majority (85.8%, *n* = 176) of undergraduates those who had sexual intercourse within last 1 year had not used condoms in their last sexual activity. Out of 17 undergraduates that had sexual intercourse with commercial sex worker, 58.8% (*n* = 10) had used condom at their last sexual activity. The percentage used condoms at last sex with non-commercial sex partners were 12.4% (*n* = 24).

### Factors associated with RSB in bivariate analysis

Being a male undergraduate and belonging to Sinhala ethnicity showed significant association with RSB in bivariate analysis. As shown in Table 2, RSB for last 3 months was negatively associated with engaging more religious activities and considering religion as more important to their lives. Having to the opportunity to talk with parents and siblings regarding sexual problems showed negative association with RSB.

**Table 2:** Unadjusted odds ratios for association of risky sexual behavior with socio-demographic, social and economic factors

Factors	Risky sexual behavior		OR	*p* value
	Yes No (%)	No No (%)	(95%CI)	
Sex				
Male	123 (64.7)	522 (37.8)	3.02 (2.20–4.15)	< 0.001
Female	67 (35.3)	859 (62.2)	Reference	
Age				
≤ 22 yrs	55 (29.3)	414 (30.0)	1.04 (0.74–1.45)	0.83
> 23 yrs	133 (70.7)	964 (70.0)	Ref	
Residence				
Outside home	137 (72.1)	948 (68.5)	1.19 (0.85–1.66)	0.31
In home	53 (27.9)	436 (31.5)	Ref	
Religion				
Buddhist	176 (92.6)	1238 (89.4)	1.50 (0.84–2.64)	0.17
Non-Buddhists	14 (7.4)	147(10.6)	Ref	
Ethnicity				
Sinhala	188 (98.9)	1292 (93.3)	6.77 (1.65–27.69)	0.002
Non- Sinhala	2 (1.1)	93 (6.7)	Ref	
Income				
≤ 50,000 rupees	139 (77.2)	1055 (82.8)	0.70 (0.48–1.03)	0.07
> 50,000 rupees	41 (15.8)	219 (17.2)	Ref	
Financial support				
> 3000 Rs/month	13 (9.8)	81(7.8)	1.28 (0.69–2.37)	0.43
≤ 3000 Rs/month	119(90.2)	951 (92.2)	Ref	
Academic year				
Second	112 (58.9)	818 (59.1)	1.005(0.74–.37)	0.98
Third	78 (41.1)	567 (40.9)	Ref	
Academic stream				
Bio-Science	26 (13.7)	222 (16.0)	0.83 (0.54–1.29)	0.41
Non Bio-Science	164 (86.3)	1163 (84.0)	Ref	
School type				
Mixed	70 (49.0)	575 (51.8)	0.89 (0.63–1.26)	0.51
Non-mixed	73 (51.0)	534 (48.2)	Ref	
Religious activities				
More	58 (30.5)	615 (44.8)	0.54 (0.39–0.75)	< 0.001
Less	132 (69.5)	759 (55.2)	Ref	
Importance of religion				
More	118 (62.1)	1084 (78.3)	0.46 (0.33–0.63)	< 0.001
Less	72 (37.9)	301 (21.7)	Ref	
Access to talk with relative,				
Yes	110 (57.9)	969 (70.1)	0.59 (0.43–0.80)	0.001
No	80 (42.1)	416 (30.0)	Ref	
Access to talk with friends				
Yes	177 (93.2)	1231 (88.9)	1.70 (0.95–3.06)	0.07
No	13 (6.8)	154 (11.1)	Ref	

Further, RSB were significantly associated with undergraduates who attended nightclubs more than once within last month, those who had used internet facilities > 2 h per day, those who went to cinema ≥2 per months, who had taken alcohol within last 3 months, who had smoked within last 3 months, who had taken ganja (cannabis) within last 3 months and who had physical fight within last 1 year in university (Table 3).

**Table 3:** Unadjusted odds ratio for association of risky sexual behaviors with other risk behaviors

Other risk behavior	Risky sexual behavior		OR	*p* value
	Yes No (%)	No No (%)	(95%CI)	
Attend night clubs in last month				
≥ once /month	9 (4.7)	8 (0.6)	8.56 (3.26–22.46)	
Not in last month	181 (95.3)	1377 (99.4)	Ref	< 0.001
Using Internet facilities				
≥ 2 h/day	88 (46.3)	526 (38.0)	1.41(1.04–1.91)	
< 1 h/day	102,953.7)	859 (62.0)	Ref	0.027
Monthly frequency of going to cinema halls				
≥ 2per month	34 (17.9)	134 (9.7)	2.03 (1.35–3.07)	0.001
< 1 per month	156 (82.1)	1251(90.3)	Ref	
Had taken alcohol in last three months				
Yes	79 (41.6)	195 (14.1)	4.34 (3.14–6.02)	< 0.001
No	111 (58.4)	1190 (85.9)	Ref	
Had smoked within last three months				
Yes	33 (17.4)	76 (5.5)	3.62 (2.33–5.63)	< 0.001
No	157 (82.6)	1309 (94.5)	Ref	
Had taken Marijuana within last three months				
Yes	15 (7.9)	32 (2.3)	3.62 (1.92–6.83)	
No	175 (92.1)	1353 (97.7)	Ref	< 0.001
Had physical fighting in last one year in university				
Yes	25 (13.2)	35 (2.5)	5.84 (3.41–10.01)	
No	165 (86.8)	1350 (97.5)	Ref	< 0.001

Good knowledge on contraceptives, good knowledge on condoms, good knowledge on sexual and RH rights, good overall knowledge on RH, favorable attitudes on contraceptives, favorable attitudes on condoms and favorable overall attitudes on RH showed statistically significant associations in bivariate analysis with RSB (Table 4).

**Table 4:** Unadjusted odds ratios for association of RSB with knowledge and attitudes on reproductive health

Knowledge and attitude aspect	Risky sexual behavior		OR	*p* value
	Yes No (%)	No No (%)	(95%CI)	
Knowledge on Unsafe abortions				
Good	109 (57.4)	730 (52.7)	1.21 (0.89–1.64)	
Average^a^	81 (42.6)	655 (47.3)	Ref	0.227
Knowledge on Contraceptives				
Good	71 (37.4)	376 (27.1)	1.60 (1.17–2.0)	
Average^a^	119 (62.6)	1009 (72.9)	Ref	0.003
Knowledge on Condoms				
Good	145 (76.3)	597 (43.1)	4.25 (3.00–6.04)	< 0.001
Average^a^	45 (23.7)	788 (56.9)	Ref	
Knowledge on Sexually Transmitted Infections				
Good	35,918.4)	227 (16.4)	1.15 (0.78–1.71)	0.481
Average^a^	155 (81.6)	1158 (83.6)	Ref	
Knowledge on HIV/AIDS				
Good	125 (66.8)	851(62.0)	1.23 (0.89–1.71)	0.201
Average^a^	62(33.2)	521 (38.0)	Ref	
Knowledge on Sexual and Reproductive health rights				
Good	58 (30.5)	327 (23.6)	1.42 (1.02–1.98)	
Average^a^	132 (69.5)	1058 (76.4)	Ref	0.038
Overall knowledge on Reproductive health				
Good	45 (23.7)	241 (17.4)	1.47 (1.03–2.12)	0.035
Average^a^	145(76.4)	1144 (82.6)	Ref	
Attitude on contraceptives				
Desirable	75 (39.5)	402 (29.2)	1.58 (1.15–2.16)	0.004
Undesirable	115 (60.5)	974 (70.8)	Ref	
Attitude on Condoms				
Desirable	121 (63.7)	487 (35.4)	3.20 (2.33–4.38)	< 0.001
Undesirable	69 (36.3)	888 (64.6)	Ref	
Attitude on HIV/AIDS				
Desirable	93 (49.5)	588 (42.7)	1.32 (0.97–1.78)	0.081
Undesirable	95 (50.5)	788 (57.3)	Ref	
Attitude on Overall Reproductive health				
Desirable	92 (48.4)	388 (38.0)	2.41 (1.77–3.28)	< 0.001
Undesirable	98 (51.6)	997 (72.0)	Ref	

^a^Satisfactory and poor knowledge were amalgamated as average knowledge

OR, odds ratio, CI, confidence interval

### Factors associated with RSB in multivariate analysis

Multiple logistic regression model included 1575 participants. RSB during the last 3 months showed significant association with four factors. Those who had taken alcohol within last 3 months (AOR 2.59, 95% CI: 1.82–3.70), those who had attended nightclub more than once in last month (AOR 3.61; 95% CI: 1.31–9.97), and those who had good knowledge on condoms (AOR 2.91, 95% CI: 2.00–4.24), showed positive association with RSB. Those who considered the religion to be important to their lives (AOR 0.67, 95% CI: 0.48–0.95), showed negative association with RSB. (Table 5) Reanalysis of data excluding the variable ‘use of alcohol within last three months’ had not changed the results.

**Table 5:** Adjusted odds ratios for Risky Sexual Behavior among undergraduates within last three months

Variable	β co-efficient	SE	OR	95% CI	*p* value
Had taken alcohol within last three months	0.951	0.18	2.59	1.82–3.70	< 0.001
Attended night clubs more than once in last month	1.284	0.52	3.61	1.31–9.97	0.013
Good knowledge on condoms	1.069	0.19	2.91	2.00–4.24	< 0.001
Considered religion was more important to their lives	−0.399	0.175	0.67	0.48–0.95	0.022

Hosmer and Lemeshow Test Chi-square value 3.8, *p* value 0.43

SE, standard error, OR, odds ratio, CI, confidence interval

## Discussion

In this study an attempt has been made to assess the prevalence of RSB and factors associated to RSB among undergraduate students in state universities of Western Province in Sri Lanka.

### Prevalence of RSB

The prevalence of RSB was 12.4 and 12.1% for last 1 year period and for last 3 months period respectively. Prevalence among male and female undergraduates were 19.1 and 7.2% respectively. A study carried out among first year Agricultural undergraduates of University of Ruhuna, Sri Lanka reported that 2% had multiple sexual partners during last 3 months which was considered as a risk behavior [28]. In the present study, having multiple partners during last 3 months was 5% and the observed difference may be due to the difference of the sample selected i.e. second and third year representing all study streams from four universities. Another study carried out among Ethiopian undergraduates revealed that 18.6% of them had lifetime multiple sexual partners which was 13.7% in the present study [29]. Their use of condoms during first sexual intercourse was 11.2%, much lower than the present 18% figure. Their figure for not using condoms within the last 1 year period was 39.4% from those who had sex in last 1 year. In the present study, not using condoms in last sexual activity was 85.8% which was much higher. The figure may be due to the reason that our young people are having “somewhat mutually monogamous” relationships which they would think the need of condom use is unnecessary.

### Factors associated with RSB

In the present study, those who had good knowledge on condoms showed a positive association with RSB. This finding was in line with a study carried out in Washington which discussed an association with knowledge on condoms and condom usage. Failure to use protective method in risk behavior may not be due to the ignorance but may be the inability of perceiving the risk [30]. Undergraduates who had experienced or are interested in sexual behaviors may be enthusiastic in finding more information on preventive measures of STI i.e. usage of condoms. Peltzer has stated that those who had recent sexual exposures had correct knowledge on condoms [31].

The present study revealed that those who considered religion is more important to their lives were less likely to be associated with RSB than those who did not. Similar comparable results were also reported among adolescent and young adults in USA [22]. In contrast to the findings, a study done among undergraduates at the University of Kentucky revealed that students with higher religious beliefs but lower religious behaviors were at risk for risky sexual practices [32].

Frequently attending nightclubs showed significant positive association with RSB in the present study. In compatible with these findings, attending night clubs showed significant association with having sex ever, having multiple sexual partners and having sex with commercial sex workers in a study from Ethiopia [33]. The difference in the degree of association may be due to the different definitions of RSB in these two studies. Difference of academic year of selected study participants may have contributed to the observed dissimilarity. The Ethiopian study had selected undergraduates from all 5 years including 1st, 4th and 5th academic years which we have excluded in the present study. Another study conducted among undergraduates had showed different degree of positive association between RSB and attending night clubs [11].

Present study showed a statistically significant positive association with alcohol consumption within the past 3 months’ time. The results were compatible with few other studies [16, 25, 34, 35]. Alcohol use together with sexual activities itself are within the definition of RSB in our study, even though none of them had taken alcohol intake as a factor in their definition of RSB. The observed association may be due to an impaired decision making ability and dis-inhibition behavior due to alcohol consumption.

Kebede et al. revealed a positive association between unprotected sex and using alcohol daily [20]. Alcohol consumption has been measured for past 3 months’ time while unprotected sex for the period of last 1 year. Undergraduates in England perceived that life style in university provided opportunities for risky sex via high level of alcohol consumption along with other factors like increased sexual opportunities [35]. As described by Cooper, drinking alcohol may have an association that could not be described easily [21]. Therefore there exists a definite need for further research, both quantitative and qualitative in order to describe relationship between alcohol consumption and RSB.

Given the cross sectional nature of the study design, it was difficult to identify cause and effect association between the variables. As the discussed topic was very sensitive and the information was self-reported, there may be possibility of deliberately hiding of information in relation to unacceptable behavior. The results could be generalized to all the universities in Western Province and all university students in Sri Lanka as participants for the study are from all over the country.

## Conclusions

Risky sexual behavior was prevailing among undergraduates at a rate of 12.4%. Males had more RSB than females. Those who had taken alcohol within last 3 months, had attended night clubs more than once in the last month and had good knowledge on condoms were associated with higher risk of RSB Undergraduates’ consideration of the religion as more important to their lives had lower risk with RSB.

Authorities of university and health care providers should consider the need and take necessary actions to establish accessible, affordable RH services within university. They should consider taking necessary steps to minimize alcohol consumption within university and outside society. Authorities within university and outside should discourage night clubs attendance among undergraduates by encouraging more recreational activities with the help of peer leaders, academic and non-academic staff members and other organizations. Religious activities should be promoted within universities and outside.

## Abbreviations

AIDS: Acquired immuno deficiency syndrome
CDC: Centers for disease control and prevention
CI: Confidence interval
HIV: Human immuno-deficiency virus
OR: Odds ratio
PPS: Probability proportionate to size
RH: Reproductive health
RSB: Risky sexual behaviors
SPSS: Statistical package for social science
STI: Sexually transmitted infections

## References

[1] Brener ND, Kann L, Kinchen SA, Grunbaum JA, Whalen L, Eaton D, et al. Methodology of the youth risk behavior surveillance system. Morb Mortal Wkly Rep. 2004;53(RR-12):1–13. 15385915

[2] Madise N, Zulu E, Ciera J. Is poverty a driver for risky sexual behavior? Evidence from National Surveys of adolescents in four African countries. Afr J Reprod Health. 2007;11(3):83–98. 20698061

[3] Abels MD, Blignaut RJ (2011). Sexual risk behavior among sexually active first year students at the University of the Western Cape, South Africa. Afr J AIDS Res.

[4] Averett S, Corman H, Reichman NE. Effects of Overweight on Risky Sexual Behavior of Adolescent Girls. https://www.nber.org/papers/w16172. Accessed 24 Dec 2012.

[5] Silas J. Poverty and Risky Sexual Behaviors: Evidence from Tanzania, ICF International Calverton, Maryland, USA. 2013. pubs/pdf/WP88/WP88.pdf. Accessed 10 Dec 2015.

[6] Fernando NS. Sexual behavior and substance abuse among youth in the coastal region in Galle district. Thesis (MD in Community medicine, Postgraduate Institute of Medicine, University of Colombo). 2009.

[7] Thalagala N, Rajapaksha L (2004). National survey on emerging issues among adolescents in Sri Lanka. UNICEF.

[8] Perera B, Reece M (2006). Sexual behavior of young adults in Sri Lanka: implications for HIV prevention. AIDS Care.

[9] Sujay R (2009). Premarital Sexual Behavior among Unmarried College Students of Gujarat, India. Health and Population Innovation Fellowship Programme, Working paper.

[10] Raj RP, Padam S, Edwin RT (2010). There are too many naked pictures found in papers and on the net : factors encouraging premarital sex among young people of Nepal. Health Sci J.

[11] Dingeta T, Oljira L, Assefa N (2012). Patterns of sexual risk behavior among undergraduate university students in Ethiopia: a cross-sectional study. Pan Afr Med J.

[12] Tura G, Alemseged F, Dejene S. Risky sexual behavior and predisposing factors among students of Jimma University, Ethiopia. Ethiop J Heal Sci. 2012;22(3):170–80. PMC3511895 23209351

[13] Musiime KE, Mugisha JF. Factors associated with sexual behaviour among students of Uganda martyrs university. Int J Public Health Res. 2015;3(1):1–9. www.openscienceonline.com/author/download?paperId=1347&stateId=8000. Accessed 10 Jan 2016.

[14] Soboka B, Kejela G. Assessment of Risky Sexual Behaviors among Arba Minch University Students, Arba Minch Town, Snnpr, Ethiopia. J Child Adolesc Behav. 3(2). 10.4172/2375-4494.1000189. Accessed 17 Jan 2016.

[15] Mavhandu-Mudzusi, AH, Asgedom T. The prevalence of risky sexual behaviours amongst undergraduate students in Jigjiga University, Ethiopia Health Sagesoheid. 2016;21 (17):179–86. www.scielo.org.za/pdf/hsa/v21n1/56.pdf. Accessed 31 Mar 2016.

[16] Wordofa D, Shiferaw S. Sexual risk behaviors and its associated factors among undergraduate students in Madda Walabu university, Southeast Ethiopia: a facility based cross sectional study. Epidemiology. 10.4172/2161-1165.1000207. Accessed 30 Apr 2016.

[17] Bayissa D, Mebrahtu G, Bayisa G, Mekuanint Y. Assessment of Early Sexual Initiation and Associated Factors among Ambo University Undergraduate Students, Ambo, Ethiopia. J Health Med Nurs. 2016;25:35-40.

[18] Ruangkanchanasetr S, Plitponkarnpim A, Hetrakul P, Kongsakon R (2005). Youth risk behavior survey: Bangkok, Thailand. J Adolesc Health.

[19] Cooper ML (2002). Alcohol use and risky sexual behavior among college students and youth: evaluating the evidence. J Stud Alcohol.

[20] Kebede D, Alem A, Mitike G, Enquselassie F, Berhane F, Abebe Y (2005). Khat and alcohol use and risky sex behavior among in-school and out-of-school youth in Ethiopia. BMC Public Health.

[21] Cooper ML (2006). Does drinking promote risky sexual behavior? A Complex Answer to a Simple Question. Drinking Risk Sexual Behav.

[22] Haglund KA, Fehring RJ. The Association of Religiosity, Sexual Education, and Parental Factors with Risky Sexual Behaviors among Adolescents and Young Adults, Nursing Faculty Research and Publications Nursing. https://epublications.marquette.edu/nursing_fac/3/. Accessed 16 Aug 2012. 10.1007/s10943-009-9267-5 19565334

[23] http://www.ugc.ac.lk/en/statistics/university-statistics-2012.html. Accessed 17 Feb 2013.

[24] Center for Research on Environment, Health and Population Activities. Determining an Effective and Replicable Communication Based Mechanism for Improving Young Couples’ Access to and Use of Reproductive Health Information and Services in Nepal - An Operations Research Study. http://citeseerx.ist.psu.edu/viewdoc/download;jsessionid=9FE1397D0F6752DF473913172427D3A3?doi=10.1.1.175.7549&rep=rep1&type=pdf. Accessed 10 Dec 2015.

[25] Martiniuk ALD, Steel O’Connor K, King WD (2003). A cluster randomized trial of a sex education program in Belize, central America. Int J Epidemiol.

[26] Rawstorne P, Worth H. Sri Lanka Behavioural Surveillance Survey: First Round Survey Results 2006–2007, Colombo Ministry of Healthcare and Nutrition, Sri Lanka. http://www.aidscontrol.gov.lk/images/pdfs/hiv_data/2006_2007_BSS_Report.pdf. Accessed 17 Feb 2013.

[27] Cleland J. Illustrative Questionnaire for Interview-Surveys with Young People http://www.who.int/reproductivehealth/topics/adolescence/questionnaire. Accessed 21 Jan 2013.

[28] Somaratna WA. Study on knowledge and attitudes on HIV/AIDS and current sexual practices among first year agriculture students of university of Ruhuna, Dissertation. 2010 (Diploma in Reprod Health, Postgraduate Institute of Medicine, University of Colombo).

[29] Henok A, Kassa A, Lenda A, Nibret A, Lamaro T. Knowledge, attitude and practice of risky sexual behavior and condom utilization among regular students of Mizan-Tepi university, South West Ethiopia Journal of child and adolescent behavior 10.4172/2375-4494. Accessed 25 May 2016.

[30] Morrison DM, Baker SA, Gillmore MR (1994). Sexual risk behavior, knowledge and condom use among adolescents in juvenile detention. J Youth Adolescence.

[31] Peltzer K (2001). Knowledge and practice of condom use among first year students at University of the North. Curationis.

[32] Prassel HB. The influence of religiosity on risky patterns of drug usage and sexual practices in underage undergraduate students. Theses and Dissertations--Psychology. 2016;102. https://uknowledge.uky.edu/psychology_etds/102.

[33] Mulu W, Yimer M, Abera B. Sexual behaviors and associated factors among students at Bahir Dar University: a cross sectional study. Reprod Health. 2014;11(84). 10.1186/1742-4755-11-84. 10.1186/1742-4755-11-84 PMC4271440 25481831

[34] Fentahum N, Mamo A (2014). Risky sexual behaviors and associated factors among male and female students in Jimma zone preparatory schools, south West Ethiopia: comparative study. Ethiop J Health Sci.

[35] Chanakira E, O’Cathain A, Goyder EC, Freeman JV. Factors perceived to influence risky sexual behaviours among university students in the United Kingdom: a qualitative telephone interview study. BMC Public Health. 2014. 10.1186/1471-2458-14-1055. 10.1186/1471-2458-14-1055 PMC4203964 25300195

